# Does injury pattern among major road trauma patients influence prehospital transport decisions regardless of the distance to the nearest trauma centre? – a retrospective study

**DOI:** 10.1186/s13049-019-0593-7

**Published:** 2019-02-13

**Authors:** Helen Fagerlind, Lara Harvey, Stefan Candefjord, Johan Davidsson, Julie Brown

**Affiliations:** 1 Neuroscience Research Australia, 139 Barker Street, Randwick, Sydney, NSW 2031 Australia; 2 School of Medical Sciences, University of New South Wales, Sydney, NSW 2031 Australia; 3 School of Public Health and Community Medicine, University of New South Wales, Sydney, NSW 2031 Australia; 4 Division of Vehicle Safety, Chalmers University of Technology, 412 96 Gothenburg, Sweden; 5 Department of Electrical Engineering, Chalmers University of Technology, 412 96 Gothenburg, Sweden; 6 SAFER – Vehicle and Traffic Safety Centre at Chalmers University of Technology, 402 78 Gothenburg, Sweden; 7 MedTech West, Sahlgrenska University Hospital, 413 45 Gothenburg, Sweden

**Keywords:** Major trauma, Undertriage, Injury pattern, Trauma system, Road traffic injury

## Abstract

**Background:**

Prehospital undertriage occurs when the required level of care for a major trauma patient is underestimated and the patient is transported to a lower-level emergency care facility. One possible reason is that the pattern of injuries exceeding a certain severity threshold is not easily recognizable in the field. The present study aims to examine whether the injury patterns of major road trauma patients are associated with trauma centre transport decisions in Sweden, controlling for the distance from the crash to the nearest trauma centre and other patient characteristics.

**Methods:**

The Swedish Traffic Accident Data Acquisition (STRADA) database was queried from April 2011 to March 2017. Teaching hospitals with neurosurgery capabilities were classified as trauma centres (TC), all other hospitals were classified as other emergency departments (ED). Injury Severity Score ≥ 13 was used as the threshold for major trauma. Ten common injury patterns were derived from the STRADA data; six patterns included serious neuro trauma to the head or spine. The remaining four patterns were: other severe injuries, moderate to serious abdomen injuries, serious thorax injuries and all other remaining injury patterns. Logistic regression was used to analyse the effect of injury patterns, age, sex and distance from crash to nearest TC on transport decision (TC or ED).

**Results:**

Of the 2542 patients, 38.0% were transported to a TC, equating to a prehospital undertriage of 62%. Over half (59.4%) of the patients had four or more Abbreviated Injury Scale (AIS) 2+ injuries. After controlling for age, sex and distance to nearest TC, only patients sustaining serious head injuries together with other severe injuries had significantly higher odds of being transported to a TC (OR = 4.18, 95% CI: 2.03, 8.73). The odds of being transported to a TC decreased by 5% with every kilometre further away the crash location was to the nearest TC.

**Conclusion:**

These results highlight that there is considerable prehospital undertriage in Sweden and suggest that distance to nearest TC is more influential in transport decisions than injury pattern. These results can be used to further develop prehospital transportation guidelines and designation of trauma centres.

## Background

In 2016, 195 million people were injured in road transport globally [1]. Road trauma is not only a threat to life, but if survived often has a large impact on peoples’ daily life [2–4]. As emphasized in the Global Plan for the Decade of Action for Road Safety 2011–2020, improved post-crash response and emergency care is critical for achieving reductions in the burden of road traffic injuries [5].

Major trauma is any injury that is life-threating or has potential to result in life-long disability. A commonly used threshold for major trauma among registry studies has been defined as an Injury Severity Score (ISS) > 15 [6]. However, following the revision from the Abbreviated Injury Scale (AIS) 1990, update 1998 dictionary [7] (AIS98) to the AIS 2005, update 2008 dictionary [8] (AIS08), it has been recommended that the threshold for major trauma based on injuries coded to AIS08 should be ISS ≥ 13 [9]. The ISS [10] is calculated as the sum of the squares of the highest AIS code in each of the three most severely injured ISS body regions [8].

It is important that each individual trauma patient is given optimal prehospital care and is transported to and treated at an emergency care facility whose capabilities match the patient’s needs. For best outcomes, patients that need high levels of care should be taken to those facilities that can provide the required care, even if it means bypassing other emergency departments (ED) [11, 12].

Prehospital undertriage occurs when the required level of care for a severely injured patient is underestimated and the patient is transported to a lower-level trauma centre (TC) or other emergency care facility. The American College of Surgeons Committee on Trauma has suggested the optimal benchmark for undertriage of major trauma is 5%, using the threshold of ISS > 15 [13]. There are a number of reasons why higher than suggested levels of undertriage based on ISS might occur. One could be that guidelines for field triage and patient destination often include measurements of physiological parameters in combination with anatomical criteria, and these measurements are not incorporated in the ISS [14, 15]. Another possible reason is that the pattern of injuries leading to an ISS beyond a certain threshold is not easily recognizable in the field. Additional reasons might be the lack of trauma destination policies, or simply that the distance from the crash to the nearest TC is too far.

In a previous study from Sweden it was found that 62% of major trauma patients from road crashes were not directly transported to a TC [16], and thus the undertriage rate appears to far exceed the 5% benchmark suggested. One hypothesis was that inability to identify the full extent of the trauma in the field may contribute to the apparent high undertriage. Another hypothesis was that the distance from the crash to the nearest trauma centre is a major contributor to the transport decision [16]. The present study aims to examine whether the injury patterns of major road trauma patients are associated with trauma centre transport decisions in Sweden, controlling for the distance from the crash to the nearest trauma centre and other patient characteristics.

## Methods

### Data selection

The Swedish Traffic Accident Data Acquisition (STRADA) is the national information system for road crashes administrated by the Swedish Transport Agency. Two sources report information directly into the system, the police and the emergency department at hospitals and trauma centres. Individuals are matched using date, time and location of the crash along with a personal identifier, creating a comprehensive road crash data source in Sweden. The information system and procedures are explained elsewhere [17].

**Fig. 1:**
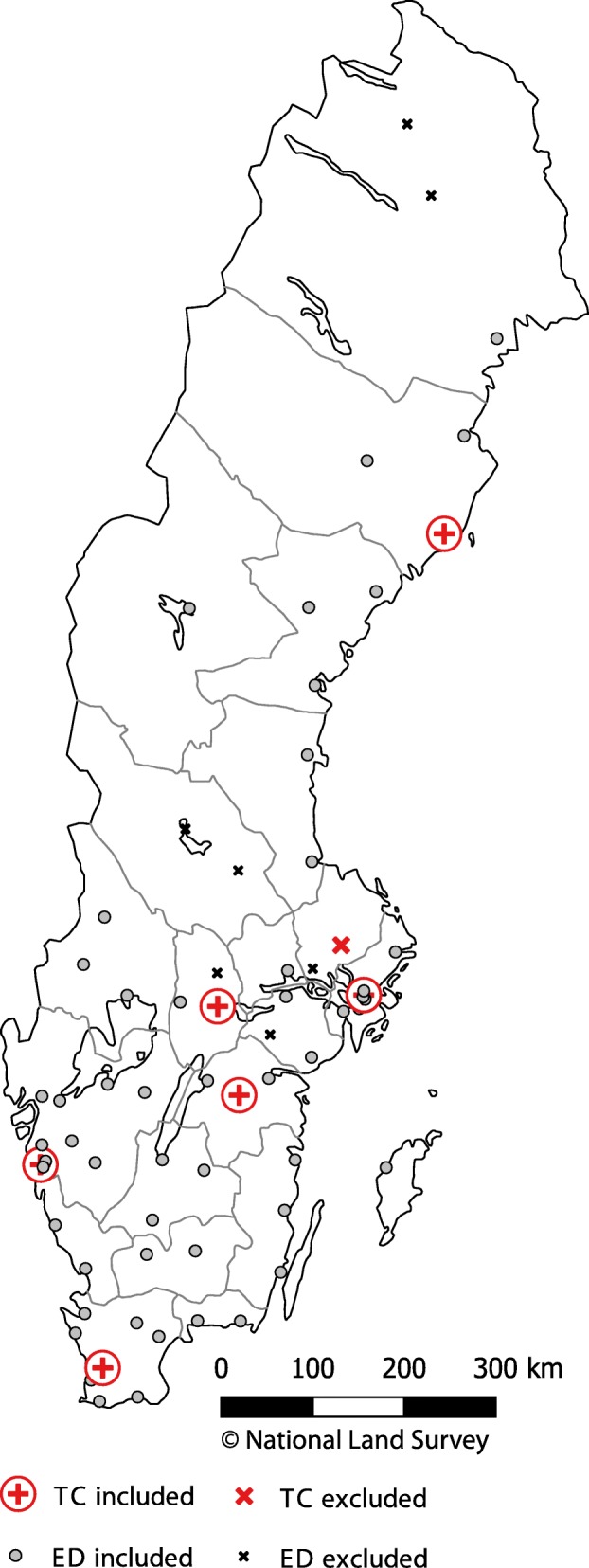
Geographical location of included and excluded trauma centres (TC) and emergency departments (ED). One of the TC symbols represent two TC’s, adult and paediatric. (This map was created in the open source software QGIS v. 3.4.3 [www.qgis.org] with open geodata from the Swedish National Land Survey [www.lantmateriet.se])

STRADA was queried from April 2011 to March 2017. The time period was chosen to optimise data coverage as data collection commenced at one TC in April 2011. In the absence of a Swedish definition of a TC, the American College of Surgeons (ACS) guidelines [13] were as used to assess the TC level. The teaching hospitals in Sweden were designated as TC’s because they fulfil the criteria for available medical resources (e.g. neurosurgery capabilities) and operates 24 h per day all year around in accordance to a Level I TC. However, the minimum requirement for Level I caseload is not met by any TC, why the designated TC’s are considered to be Level I-II. All other hospitals have lower capacity than Level II and were designated as emergency departments (ED) similar to a previous study [16]. TC’s and ED’s were included if they reported to STRADA throughout the selected time period, resulting in seven TC’s and 54 ED’s. This selection excludes one TC and seven ED’s that began reporting at a later date. Figure 1 shows the location of hospitals reporting to STRADA. Road users sustaining major trauma in the road environment (excluding pedestrian falls) who were transported from the crash scene to a hospital by ground or air ambulance were included in the study. Emergency medical services in Sweden mainly utilise nurse manned ground ambulances and roughly one third of the country is covered by anaesthesiologist manned Helicopter Medical Emergency Services (HEMS). Regional council dispatch centres decides according to their HEMS criteria if ground or helicopter ambulances should be dispatched, however, it is up to the HEMS physician to make the final decision. As injuries in STRADA are coded according to the AIS08 dictionary [8], ISS ≥ 13 was used as the threshold for major trauma. In-hospital fatalities (ISS ≥ 4) were included in the sample. ISS ≥ 13 requires at least one AIS 3 injury and one AIS 2 injury in two different ISS body regions, or at least one AIS 4 injury.

### Classification of injury patterns

The derivation of injury patterns in this study followed a similar approach used by Gabbe et al. [2] Injury patterns were first based on the Maximum AIS (MAIS) in each body region and later collapsed into fewer patterns based on body region, severity and frequency of occurrence in the dataset. Minor injuries (AIS 1) and injuries with unknown severity (AIS 9) were not included as they have little impact on the overall ISS. Therefore, injuries (AIS > 1) were extracted and divided into nine body regions; 1–8 according to the AIS dictionary (head, face, neck, thorax, abdomen, spine, upper extremities and lower extremities), and a ninth group that included all external injuries according to the ISS method. The MAIS 2–6 injuries for each of nine body regions were extracted for each individual. This approach generated 835 patterns, where 550 patients had a unique pattern.

The number of injury patterns were then further reduced into ten groups (Table 1). Due to small numbers of neck injuries, these were grouped with head injuries in accordance with the ISS body region. Since the TC definition depends on whether or not a hospital had a neurosurgery department, injury patterns were populated with patients who sustained serious head injury (AIS 3+ in head and neck) or serious spine injury (AIS 3+) first. All other body regions were collapsed into MAIS 2, 3, or MAIS 4 and above (MAIS 4+). The MAIS 4+ level was selected because research has suggested that AIS 4+ injuries do not differ in mortality rate from AIS 5+ injuries [18]. The pattern *Other 4+* injuries was selected after the neuro trauma patterns since they likely fulfil the criteria for highest triage level due to massive bleeding or major fractures. *Abdomen injuries of 2 or 3 with other injuries* was kept separate since it was assumed that this group might have sustained occult injuries not recognisable in the field [19]. The last specific injury pattern was *Thorax 3 with other injuries* followed by an *Other* category.

**Table 1:** Theoretical representation of injury patterns for ISS ≥ 13 or in-hospital fatalities

Injury pattern	AIS region and level of severity																							
	Head & Neck			Face			Thorax			Abdomen			Spine			Upper extremities			Lower extremities			External		
	2	3	4+	2	3	4	2	3	4+	2	3	4+	2	3	4+	2	3	4+	2	3	4+	2	3	4+
Head isolated^a^	O	O	C1																					
Head 3+ & other 4+	O	C1	C1	O	O	C2	O	O	C2	O	O	C2	O	O	C2	O	O	C2	O	O	C2	O	O	C2
Head 3+ thorax 3+ & other	O	C1	C1	O	O		O	C1		O	O		O	O		O	O		O	O		O	O	
Head 3+ face 2, 3 & other	O	C1	C1	C2	C2		O			O	O		O	O		O	O		O	O		O	O	
Head 3+ & other	O	C1	C1				C2			C2	C2		C2	C2		C2	C2		C2	C2		C2	C2	
Spine 3+ & other	O			O	O	O	O	O	O	O	O	O	O	C1	C1	O	O	O	O	O	O	O	O	O
Other 4+^b^	O			O	O	C1	O	O	C1	O	O	C1	O			O	O	C1	O	O	C1	O	O	C1
Abdomen 2, 3 & other^c^	O			O	C2		O	C2		C1			O			O	C2		O	C2		O	C2	
	C2			C2	C2		C2	C2		O	C1		C2			C2	C2		C2	C2		C2	C2	
Thorax 3 & other	C2			C2	C2		O	C1					C2			C2	C2		C2	C2		C2	C2	
Other^a,b^	C2			O	C1		C2						C2			O	C1		O	C1		O	C1	

C1 (Criterion 1) indicate injuries that had to be present for the patient to be included in that injury pattern, and C2 (Criterion 2) indicates that at least one of these injuries had to be present for the patient to be included in that injury pattern. Other possible injury (O) can be present or not

^a^Include fatalities ISS ≥ 4 with MAIS 2 or 3

^b^At least one of C1

^c^First row if AIS 2, second row if AIS 3

### Distance from crash to hospital

Distance (kilometres) between the crash to the first treating hospital and the crash to the nearest TC was analysed using the Global Positioning System (GPS) World Geodetic System (WGS 84). The crash location reported by the police was used when available, otherwise the location was taken from the hospital record. Distance was calculated by Vincety’s formulae [20] in the R Package geosphere [21] and multiplied by a circuity factor of 1.3 to account for road detours from a straight line [22]. The percentage of patients as a function of distance from the crash to nearest trauma centre for TC and ED destination was calculated according to the empirical cumulative distribution function (ECDF). The ECDF computes the proportion of patients less than or equal to a specified distance away from the crash location to the nearest TC.

### Data analysis

Binomial logistic regression was used to examine the effect of explanatory variables on the outcome of transport decision, which was either transported to a TC or transported to an ED. Patient’s characteristics included were age and sex. Injury patterns was modelled as ten categorical variables with the group with least proportion of TC transports used as reference. Distance from crash to the nearest TC was modelled as a continuous variable. Each explanatory variable was first modelled individually, then all explanatory variables were entered into the multivariable regression model. Statistical calculations were performed with R and RStudio [23, 24]. Statistical significance was evaluated using *p* < 0.05.

## Results

The sampling criteria resulted in an initial sample of 2573 major road trauma patients. The GPS position of the crash was missing for 107 individuals, of which 76 were identified through matching with other individuals in the same crash. The remaining 31 individuals with unknown crash location were excluded, resulting in a final sample of 2542 patients. There were 40.0% passenger car occupants, 22.1% cyclists, 16.0% motorcycle riders and pillion passengers, 13.2% pedestrians, 5.8% moped riders and 2.8% truck and bus occupants. Overall the median age was 50 years (interquartile range [IQR] 31–65). In total there were 38.0% (*n* = 966) patients transported to a TC by ground or air ambulance. The TC proportion for ISS < 16 was 34.1% (*n* = 308 of 904) and for ISS > 15 the TC proportion was 40.2% (*n* = 658 of 1638). About one third of females (33.3%) and 40.0% of men were taken to a TC.

After injuries with a minor or unknown severity were excluded (*n* = 4574), a total of 12,759 injuries were considered. Table 2 presents the number of AIS 2+ injuries by the number of involved AIS body regions. There were 59.4% patients with four or more AIS 2+ injuries. Multiple AIS 2+ injuries in the same body region occurred in 73.6% of patients (Table 2, shown in bold).

**Table 3:** Logistic regression models of major trauma patients ISS ≥ 13 or in-hospital fatalities (*N* = 2542)

	TC	Univariate			Multivariable		
	n (%)	OR	95% CI	*р*	OR	95% CI	*р*
Injury pattern							
Head isolated^a^	56 (43.1)	1.88	1.19, 2.98	< 0.01	1.51	0.75, 3.03	0.248
Head 3+ & other 4+	67 (57.3)	3.33	2.08, 5.37	< 0.001	4.18	2.03, 8.73	< 0.001
Head 3+ thorax 3+ & other	85 (48.6)	2.35	1.54, 3.59	< 0.001	1.63	0.88, 3.04	0.125
Head 3+ face 2, 3 & other	84 (40.4)	1.68	1.12, 2.54	< 0.05	0.82	0.45, 1.48	0.510
Head 3+ & other	108 (38.2)	1.53	1.05, 2.26	< 0.05	0.92	0.53, 1.60	0.760
Spine 3+ & other	85 (33.9)	1.27	0.86, 1.90	0.236	1.19	0.67, 2.11	0.555
Other 4+	138 (38.0)	1.52	1.06, 2.21	< 0.05	1.04	0.62, 1.77	0.871
Abdomen 2, 3 & other^b^	60 (28.7)	1.00			1.00		
Thorax 3 & other	170 (35.1)	1.34	0.95, 1.92	0.1	0.73	0.44, 1.21	0.219
Other^a^	113 (35.1)	1.34	0.92, 1.96	0.126	1.43	0.82, 2.51	0.209
Distance to nearest TC		0.95	0.95, 0.96	< 0.001	0.95	0.95, 0.96	< 0.001
Age		1.00	0.99, 1.00	0.346	1.00	0.99, 1.00	0.197
Sex							
Female^b^	250 (33.3)	1.00					
Male	716 (40.0)	1.33	1.12, 1.60	< 0.01	1.18	0.91, 1.54	0.216

^a^Include fatalities ISS ≥ 4 with MAIS 2 or 3

^b^Reference variable

**Fig. 2:**
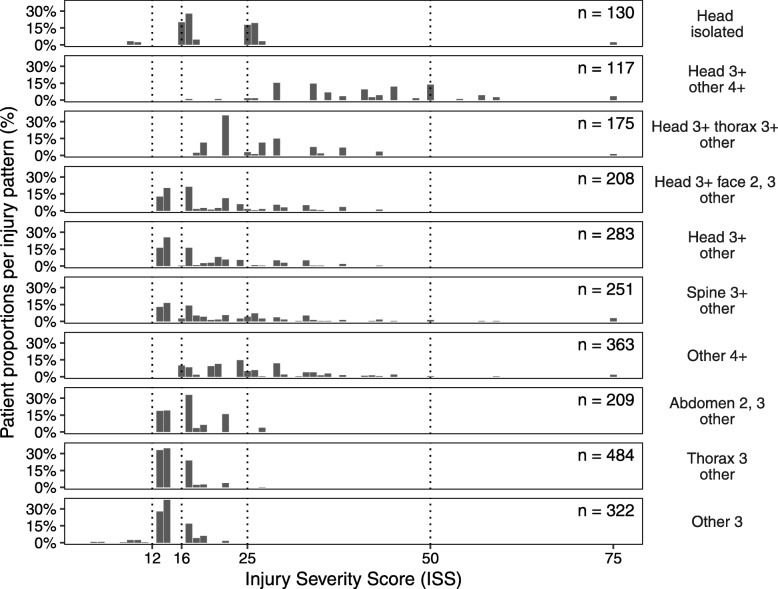
Distribution of ISS injury severity by injury pattern. ISS = 12, 16, 25, 50 and 75 are marked to distinguish important thresholds for ISS

Figure 2 illustrates the distribution of ISS injury severity for each of the ten injury patterns after allocating each patient into one of them. It shows that the majority of patients in the first eight patterns have an injury severity of ISS > 15, whereas in the last two patterns the majority of patients have an ISS < 15. Table 3 presents the proportion of patients taken to a TC for each injury pattern, where undertriage ranges from 42.7% for *Head 3+ & Other 4+* and 71.3% for *Abdomen 2, 3 and other injuries*.

**Table 2:** Number of AIS body regions injured by number of AIS 2+ injuries recorded per patient

	No. of coded AIS 2+ injuries						
No. of body regions	1	2	3	4	5	≥ 6	%
1	123	**89**	**34**	**25**	**9**	**8**	11.3
2	0	347	**326**	**181**	**92**	**95**	41.0
3	0	0	112	**154**	**137**	**269**	26.4
4	0	0	0	33	**53**	**261**	13.7
5	0	0	0	0	7	**138**	5.7
6	0	0	0	0	0	36	1.4
7	0	0	0	0	0	13	0.5
Cumulative %	4.8	22.0	40.6	56.0	67.7	100.0	

Numbers in bold represents those individuals (*n* = 1871, 73.6%) that sustained more than one injury in at least one AIS body region

**Fig. 3:**
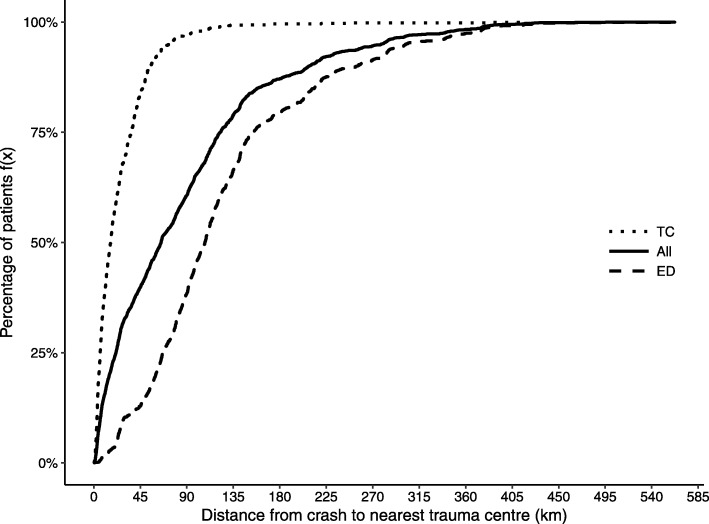
Cumulative percentage of patients as a function of the ground distance from the crash to the nearest trauma centre (TC)

The median distance from the crash to the first destination hospital was approximately 22 km (IQR 7–42). Figure 3 presents the cumulative percentage of patients as a function of the estimated ground distance from the crash to the nearest TC, for different transportation destinations. For example, about 8% of the ED transported patients were within a distance of 45 km to a TC. The median distance for all patients was approximately 65 km (IQR 21–122). For patients transported to a TC the median distance to a TC was approximately 16 km (IQR 6–36) and for patients transported to ED the median distance to the nearest TC was approximately 107 km (IQR 66–154).

Logistic regression models are presented in Table 3. Compared to the reference group of patients with pattern *Abdomen 2, 3 and other,* patients within all five groups of head injuries and *Other 4+ injuries* had significantly higher odds of being transported to a TC in the univariate analysis. When controlling for age, sex and distance to nearest TC, only patients with *Head 3+ & other 4+* had significantly higher odds (OR = 4.18, 95% CI: 2.03, 8.73) of being transported to a TC.

The odds of being transported to a TC decreased by 5% with every km further away the crash location was to the nearest TC (OR = 0.95, 95% CI: 0.95, 0.96, *p* < 0.001), and this remained unchanged in the multivariable model. Patient age was not associated with the transport decision. In the univariate analysis males had significantly higher odds (OR = 1.33, 95% CI: 1.12, 1.60) of being transported to a TC compared to females, but sex was not significant in the multivariable model.

## Discussion

This study demonstrated differences in transport decisions between major road trauma patients with different patterns of injury after controlling for age, sex and the distance to nearest TC. Patients that sustained *Head 3+ & other 4+* injuries had significantly higher odds of being transported to a TC than those who sustained the *Abdomen 2, 3 and other* pattern. Whilst this result is not unexpected as the *Head 3+ & other 4+* pattern includes those with the most severe injuries (Fig. 2), just under half (42.7%) of these patients were not transported to a TC, highlighting considerable undertriage in this high-risk group. Using our threshold of ISS ≥ 13, the overall undertriage was 62.0% in the present study. When using the higher threshold of ISS > 15 the undertriage was 59.8% in the present study compared to 62.0% previously published by Candefjord et al. [16] who used ISS > 15 as their threshold for major trauma. However, this difference was not statistically significant, which indicates that the practice norms for prehospital trauma patients are largely unchanged. Their analysis was conducted with data from the same source as this study but analysed years 2007–2014 whereas we analysed April 2011 – March 2017. The ground distance between the crash and nearest TC was independently associated with transport to TC irrespective of injury pattern or other patient characteristics. This suggests that distance to TC may be an important factor in undertriage levels. Doumouras et al. [25] demonstrated that even if a TC was reachable within 30 min in an urban setting in Toronto, Canada, the compliance with the triage protocol was reduced as the differential distance increased from the crash to the TC compared to the closest hospital.

It is important to note that our data does not include the outcome of the assessment according to any prehospital physiological parameters, and therefore it remains unclear as to what extent these factors may also have influenced the transport decision. The Glasgow Coma Scale [26], respiratory values and systolic blood pressure are commonly used in field triage guidelines, and we were unable to control for these parameters. However, a recent review reported that while vital data parameters in isolation are highly specific, they have low sensitivity for identifying major trauma [27]. Based on the physiological parameters it might be more appropriate for some patients to be transported to the nearest operating room rather than to the nearest TC. Practice norms varies between regions in Sweden and even if educational requirements for emergency care responders [16] are high, intubation and chest tube insertion are generally not performed in the prehospital setting. Therefore, some patients may need to be stabilised at the nearest ED before further transfer to a TC. Generally, ground ambulances transport the patients to the closest hospital or to the dedicated ED/TC within the region. However, our results show that even when a TC is within relatively close proximity to the crash, many patients are still transported to an ED (Fig. 3). Our results reflect the overall national situation since we did not control for regional variations in our model. Therefore, some health care regions in Sweden may have lower or higher levels of undertriage than observed in our analysis.

The lack of national guidelines for a trauma system with designated TC’s in Sweden might affect the undertriage rate observed. Even if regional guidelines exist there is potential for variation across different regions. In other jurisdictions, the presence of co-ordinated trauma management systems has been shown to reduce the population burden and increase the survival rate of major trauma [2, 28, 29]. The US trauma system ranks trauma centres from Level-I to Level-V where Level-I are designed to treat the most seriously injured patients [13]. Our definition of TC is similar to Level-I or Level-II classifications, which according to the guidelines require access to neurosurgery. Some of the ED’s are operational 24 h per day all year around (similar to Level III) and some have dedicated trauma teams. These hospitals may have been the accurate decision for some of the patient injury patterns in this study, and including these hospitals as ED’s may have biased the results towards a higher undertriage level. The information required to make explicit judgements about trauma services provided in the ED hospitals was not reliably available throughout the study period and was therefore not considered in the analysis.

Our selection of the threshold for major trauma may affect the number of individuals in the sample. A strength of the data source we used is that it contains all types of road trauma and all levels of injury severity coded to the AIS08 dictionary, allowing the threshold for major trauma to be retrospectively selected. Choosing another threshold of major trauma may have altered the sample size in our study slightly. For example another approach is to use the New Injury Severity Score (NISS) [30] larger than 15 (NISS > 15) as suggested in the Utstein template [31]. The NISS calculates the three most severe injuries regardless of body region and therefore the NISS is equal to or higher than the ISS. Tohira et al. [32] suggested that NISS is better at predicting mortality of blunt trauma patients. For our sample, a NISS > 15 threshold would mean that the bars in Fig. 2 would be shifted towards the right. If we had chosen to use NISS > 15, we would have included more patients with multiple AIS 3 in the same body region and 34% of surviving patients with ISS = 13–14 would have been excluded.

The classification of injury patterns may also affect the number of patients in each group. We could not find any well described methodology on how to classify (road) trauma patients by injury patterns that account for injuries across different body regions. In Table 2, 11.3% of patients had one or several injuries in one body region, which leaves 88.7% with multiple injuries in at least two body regions. Individuals were only counted once and classified into the injury patterns in the order described in the method section and Table 1. As injuries to the central nervous system are the largest contributor to mortality from trauma [33] we prioritised head and spine injuries in our classification approach. We believe that the methodology introduced in this study to classify injury patterns may provide a new pathway for considering the full impact of injuries to major road trauma patients. However, further work is required to examine the relationship between this classification system and longer term outcomes.

There were some limitations to the study. This was a retrospective study of road crash injury data and as noted above we could not take any on-scene considerations into account. Therefore the results cannot conclude whether the prehospital decision was appropriate or not given the patients physiological status and other crash and environment factors. This analysis did not evaluate the resuscitation by medical personnel and we did not consider any transfers from ED to TC. The transportation time could not be retrieved for this analysis and therefore the ground distance from crash to nearest TC was used as a proxy. When calculating the ground distance from the crash to the nearest TC an approximation of the distance was used. The same circuity factor was applied to all distances and was based on a published value for Germany [22] in the absence of a Swedish factor. Swedish and German road network design is assumed to be similar. The multivariable model was trialled with circuity factors of 1.2, 1.3 and 1.4 and the odds ratio for the distance variables were 0.948, 0.952 and 0.956. All other model parameters and other explanatory variable results remained the same.

## Conclusions

This study confirms an apparent large degree of undertriage in major road trauma patients in Sweden and that the distance to nearest TC has significant influence on transport decisions. Conversely, the injury patterns do not seem to influence major differences in transport decisions. However, the contribution of on-scene triage protocols remains unknown. While the impact of undertriage on mortality and morbidity was not studied here, the literature suggests appropriate prehospital triage is critical for optimum patient outcomes. For this reason, the results of this study highlight the need for further examination of the organisational level practices potentially impacting prehospital transport decisions and patient outcome. Ultimately, the results of this study may be useful for further development of prehospital transportation guidelines and future designation of trauma centres.

## Abbreviations

AIS: Abbreviated Injury Scale
ECDF: Empirical Cumulative Distribution Function
ED: Emergency Department
GPS: Global Positioning System
IQR: Inter-Quartile Range
ISS: Injury Severity Score
MAIS: Maximum Abbreviated Injury Scale
NISS: New Injury Severity Score
OR: Odds Ratio
STRADA: Swedish Traffic Accident Data Acquisition
TC: Trauma Centre
WGS: World Geodetic System
